# Author Correction: Detecting anomalous citation groups in journal networks

**DOI:** 10.1038/s41598-022-19033-7

**Published:** 2022-08-29

**Authors:** Sadamori Kojaku, Giacomo Livan, Naoki Masuda

**Affiliations:** 1 Luddy School of Informatics, Computing, and Engineering, Indiana University, Bloomington, IN 47408 USA; 2 Department of Computer Science, University College London, London, WC1E 6EA UK; 3 Systemic Risk Centre, London School of Economics and Political Science, London, WC2A 2AE UK; 4 Department of Mathematics, University at Buffalo, State University of New York, Buffalo, NY USA; 5 Computational and Data-Enabled Science and Engineering Program, University at Buffalo, State University of New York, Buffalo, NY 14260-2900 USA; 6 Faculty of Science and Engineering, Waseda University, Tokyo, 169-8555 Japan

Correction to: *Scientific Reports* 10.1038/s41598-021-93572-3, published online 15 July 2021

The original version of this Article contained an error in Figure 2(a), where the x-axis labels were displayed on a yearly basis instead of a bi-yearly basis. Additionally, in panel (c) the y-axis and the legend for the ‘JCR’ bar were omitted. The original Figure 2 and accompanying legend appear below.

**Figure 2:**
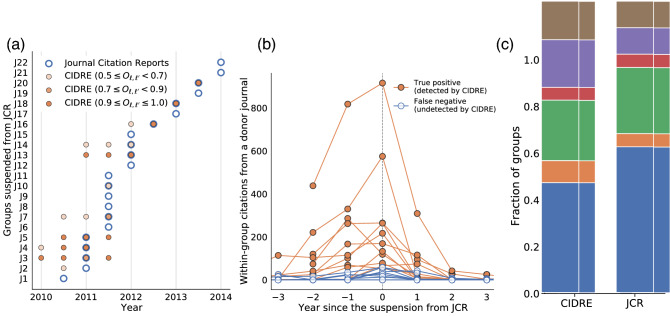
Overlap between journal groups identified by JCR and CIDRE. (**a**) Years when the journal groups are identified. The circles with thick borders represent the groups suspended from JCR. The filled circles represent those detected by CIDRE. The hue of the circles indicates the value of the overlap, $O_{\ell , \ell '}$, i.e., the fraction of journals in group $\ell '$ detected by CIDRE that belong to group $\ell$ suspended from JCR. CIDRE detected 11 out of the 22 groups suspended from JCR in the year of the suspension or before. (**b**) Number of within-group citations per year from a donor journal excluding self-citations. If the group has multiple donor journals, we show the count for the donor journal that provides the largest number of within-group citations. The horizontal axis indicates the year relative to that in which the journal group is suspended from JCR. Therefore, a negative value indicates a year before JCR suspended the journal group. For groups J17, J19, and J22, the donor journals do not provide any within-group citations for the 3 years before the suspension. (**c**) Classification of journal groups identified by CIDRE and JCR.

The original Article has been corrected.

